# The role of proprotein convertase subtilisin/kexin 9 (PCSK9) in macrophage activation: a focus on its LDL receptor-independent mechanisms

**DOI:** 10.3389/fcvm.2024.1431398

**Published:** 2024-08-01

**Authors:** Shunsuke Katsuki, Prabhash Kumar Jha, Elena Aikawa, Masanori Aikawa

**Affiliations:** ^1^ Department of Cardiovascular Medicine, Kyushu University Hospital, Fukuoka, Japan; ^2^ Center for Excellence in Vascular Biology, Cardiovascular Division, Department of Medicine, Brigham and Women’s Hospital, Harvard Medical School, Boston, MA, United States; ^3^ Center for Interdisciplinary Cardiovascular Sciences, Cardiovascular Division, Department of Medicine, Brigham and Women’s Hospital, Harvard Medical School, Boston, MA, United States; ^4^ Channing Division of Network Medicine (MA), Brigham and Women’s Hospital, Department of Medicine, Harvard Medical School, Boston, MA, United States

**Keywords:** PCSK9 (proprotein convertase subtilisin/kexin type 9), macrophages, atherosclerosis, inflammation, vascular biology, coronary artery disease, LDL receptor (LDLR), dyslipidemia

## Abstract

Recent clinical trials demonstrated that proprotein convertase subtilisin/kexin 9 (PCSK9) inhibitors reduce cardiovascular events without affecting systemic inflammation in the patients with coronary artery disease, as determined by high sensitivity C-reactive protein (CRP) levels. However, its pro-inflammatory effects in cardiovascular disease in humans and experimental animals beyond the traditional cholesterol receptor-dependent lipid metabolism have also called attention of the scientific community. PCSK9 may target receptors associated with inflammation other than the low-density lipoprotein receptor (LDLR) and members of the LDLR family. Accumulating evidence suggests that PCSK9 promotes macrophage activation not only via lipid-dependent mechanisms, but also lipid-independent and LDLR-dependent or -independent mechanisms. In addition to dyslipidemia, PCSK9 may thus be a potential therapeutic target for various pro-inflammatory diseases.

## Introduction

PCSK9, the ninth member of the proprotein convertase family, belongs to a group of serine proteases that hydrolyze peptide bonds in their cognate substrates for activation. The discovery of gain- or loss-of-function mutations in PCSK9 in patients with dyslipidemia led to the development of PCSK9 inhibitors with unprecedented lipid-lowering properties (1). PCSK9 prevents LDLR recycling to the cell surface of hepatocytes, leading to an impaired LDL-C clearance from the plasma (2). Several molecules modulate the interaction between PCSK9 and LDLR. PCSK9 interacts with heparan sulfate proteoglycans on the hepatocyte surface through the interaction of surface-exposed basic residues with trisulfated heparan sulfate disaccharide repeats, which promotes LDLR degradation (3). Cytosolic adenylyl cyclase-associated protein 1 (CAP1), a surface receptor that binds the C-terminal domain of resistin (4), also binds the CHRD of PCSK9 and enhances the degradation of the PCSK9-LDLR complex (5). Both the LDLR and PCSK9 are transcriptionally regulated by sterol regulatory element binding protein-2 (SREBP-2) (6). Mitochondrial reactive oxygen species increase PCSK9 secretion and associate with mitochondrial fission, of which dynamin-related protein 1 (DRP1) is a key driver (7). Rogers et al. of our group demonstrated that DRP1 inhibition promoted hepatic proteasomal degradation of PCSK9 to reduce hepatic PCSK9 secretion (8), by trafficking from ER to proteasomes via ER chaperone glucose-regulated protein 94 (GRP94) (9). DRP-1 inhibition also suppressed *PCSK9* mRNA levels in HepG2 cells via reduced SREBP-1c.

Pivotal studies such as the FOURIER trial (10) and ODYSSEY OUTCOMES trial (11) have demonstrated the efficacy of PCSK9 antibodies in reducing cardiovascular events in primary and secondary prevention, respectively, representing recent advancements in PCSK9-targeted intensive lipid lowering strategy. Building upon these findings, the GLAGOV (12) and HUYGENS trial (13) revealed that the LDL-C reduction by PCSK9 antibodies contributed to the stabilization of plaques as gauged by intracoronary imaging, offering a potential mechanism for their cardiovascular benefits. PACMAN-AMI trial (14) further provided evidence that an early and aggressive lipid lowering strategy with PCSK9 antibodies for high-risk patients may be beneficial. Insights from the ORION trials (15) have expanded the therapeutic landscape by indicating that a PCSK9 siRNA significantly lowers LDL-C levels. Lipoprotein (a) [Lp(a)] is a strong risk factor for atherosclerotic cardiovascular disease regardless of the reduction of LDL-C levels achieved by statins. Clinical trials such as FOURIER and ODYSSEY OUTCOMES trials demonstrated that PCSK9 inhibitors lower not only serum levels of LDL-C but also Lp(a) (16). Notably, The ODYSSEY OUTCOMES trial with alirocumab demonstrated for the first time that a reduction in Lp(a) associated with less major adverse cardiovascular events (17).

## Potential link between PCSK9 and inflammation

Genetic evidence suggests lipid-dependent effects of PCSK9 in gain-of-function and loss-of-function mutation carriers (18). Moreover, clinical trials investigating the clinical benefit of PCSK9 inhibitors using serial intracoronary imaging, such as HUYGENS (13) and PACMAN-AMI (14) trials, have revealed the relationship between achieved levels of LDL-C and change in percent atheroma volume with more intensive lipid-lowering therapies than statins or a statin plus ezetimibe (19). In addition, PCSK9 inhibitors did not change high sensitivity C-reactive protein (CRP) levels in the patients with coronary artery disease (20, 21). However, CRP may not solely capture changes in local inflammation produced by PCSK9 inhibition.

**Figure 1:**
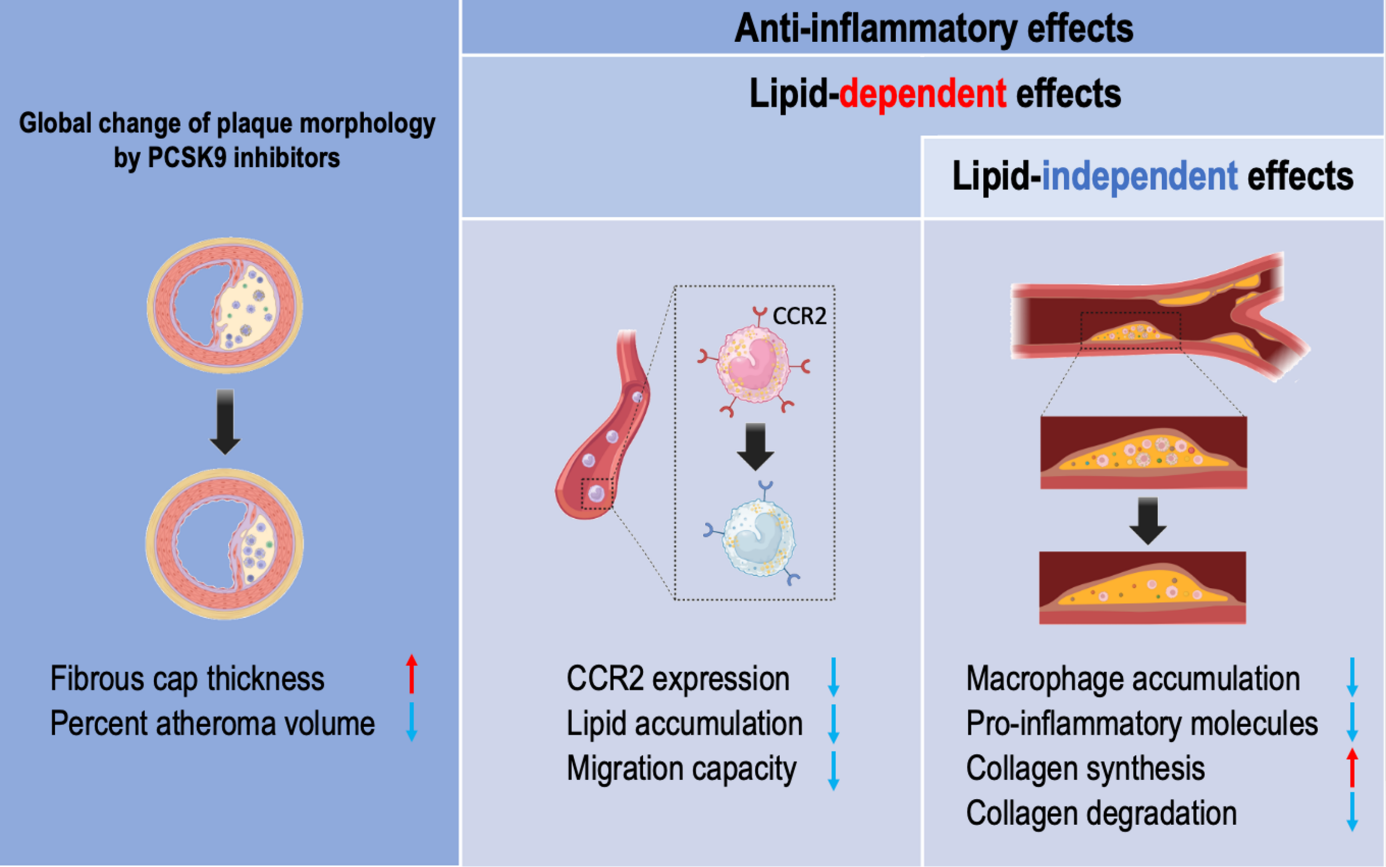
The vasculoprotective effects of PCSK9 inhibitors in humans. Lipid lowering effects of PCSK9 inhibitors include thickening of the fibrous cap thickness in coronary plaque and reduced percent atheroma volume. PCSK9 inhibitors have anti-inflammatory properties in a lipid-dependent effects and lipid-independent manner. Monocytes isolated from familial hypercholesterolemia show reduced intracellular lipid accumulation and lower CCR2 expression associated with reduced migration capacity by PCSK9 inhibitors. Patients treated with PCSK9 inhibitors have a reduced macrophage accumulation and pro-inflammatory molecules within the carotid plaque, leading to reduced collagen remodeling partially in a lipid-independent manner.

A recent clinical study showed that anti-inflammatory changes in monocyte phenotype with PCSK9 antibodies were not accompanied by a CRP reduction in patients with familial hypercholesterolemia (22). In addition, the ATHEROREMO-IVUS study (The European Collaborative Project on Inflammation and Vascular Wall Remodeling in Atherosclerosis—Intravascular Ultrasound) demonstrated a linear relationship between serum levels of PCSK9 and the fraction or amount of the necrotic core in the non-culprit lesions of patients with acute coronary syndrome, independently of serum levels of LDL-C. A recent observational study demonstrated that treatment with a PCSK9 inhibitor monotherapy is associated with a decreased major adverse cardiovascular events even after adjustment of multiple variables including LDL-C, associated with the decreased expression of inflammatory proteins within the atherosclerotic plaque (23). Collectively, these data suggested anti-inflammatory properties of PCSK9 inhibitors beyond lipid lowering (Figure 1).

**Figure 2:**
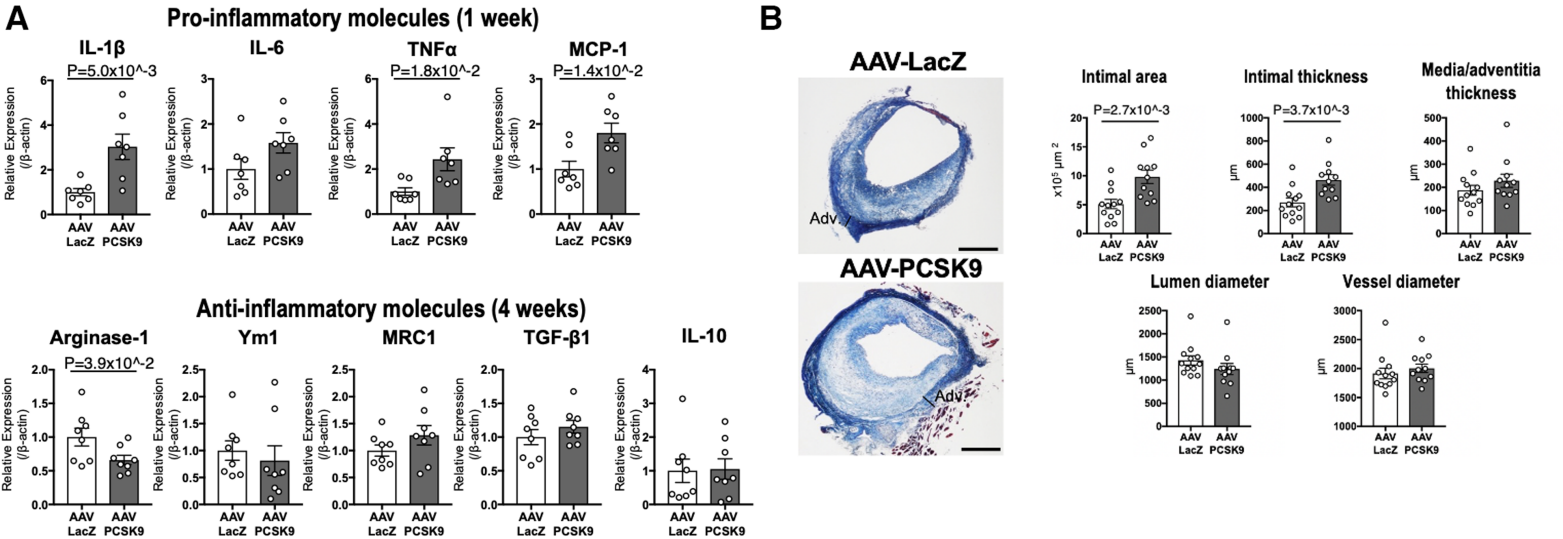
The role of circulating PCSK9 in proinflammatory macrophage activation and vein graft lesion development via LDLR-independent mechanisms. (**A**) mRNA levels of pro-inflammatory molecules and anti-inflammatory molecules were measured in murine peritoneal macrophages of *Ldlr^−/−^* mice 1 week (upper panels) or 4 weeks (lower panels) after intravenous injection with AAV-LacZ or AAV-PCSK9 (*n* = 7–8 per group). (**B**) Left panel: Masson's trichrome staining of vein grafts from *Ldlr^−/−^* mice treated with AAV-LacZ or AAV-PCSK9 28 days after implantation. Right panel: The quantitative analysis of intimal area, intimal and media/adventitia thickness, and lumen and vessel diameter of vein grafts (*n* = 12 and 11 for AAV-LacZ and AAV-PCSK9 group, respectively). Panels were reproduced from Katsuki et al. with permission of the publisher.

PCSK9 of the hepatic origin binds to LDL particles and reaches the atherosclerotic plaque via the circulating blood (24). An *in vitro* experimental study suggested the possibility that PCSK9 released by vascular smooth muscle cells in the human atherosclerotic plaque could reduce LDLR expression in macrophages (25). Considering the accumulating evidence from over decades of research that macrophages play key roles in the initiation and the development of atherosclerosis and the onset of its acute thrombotic complications, these data indicate that circulating PCSK9 and/or local PCSK9 may induce plaque inflammation via macrophage activation. Our group reported that PCSK9 induces pro-inflammatory activation of macrophages and accelerates vein graft lesion development via LDLR-independent mechanisms (Figure 2) (26). This article will provide an overview on the current evidence that links PCSK9 and inflammation via (1) lipid-dependent, (2) lipid-independent and LDL receptor-dependent, or (3) lipid-independent and LDL receptor-independent mechanisms.

## Lipid-dependent pro-inflammatory effects of PCSK9

As described above, cholesterol-lowering with PCSK9 inhibitors attenuates the pro-inflammatory profile of monocytes in patients with hypercholesterolemia. LDL-C lowering with PCSK9 antibodies reduced monocyte CCR2 expression and migratory capacity of monocytes, which was correlated with intracellular lipid accumulation in monocytes (22). This data suggested that LDL-C lowering with PCSK9 inhibitors paralleled by reduced monocyte lipid accumulation itself contributes to the anti-inflammatory effects.

In a preclinical study, weekly subcutaneous injection of alirocumab dose-dependently decreased plasma lipids (27). In the same study, alirocumab added to a statin therapy increased smooth muscle and collagen content associated with decreased macrophage accumulation and necrotic core formation in APOE*3Leiden.CETP mice (27). A strong correlation between plasma total cholesterol levels and atherosclerotic lesion area in the aortic root was observed, suggesting cholesterol lowering with a PCSK9 inhibitor itself leads to inhibition of atherosclerosis development and improves plaque morphology.

## Lipid-independent and LDL receptor-dependent pro-inflammatory effects of PCSK9

Again, PCSK9 released by vascular smooth muscle cells reduces LDLR expression in macrophages *in vitro* (25). Evidence suggests that pro-inflammatory responses to PCSK9 in macrophages and arterial atherosclerotic lesions may primarily depend on LDLR. Ricci et al. reported that recombinant PCSK9 induced a nuclear translocation of p65, a subunit of the proinflammatory transcription factor NF-κB, in macrophage-like THP-1 cells and pro-inflammatory cytokines and chemokines in THP-1 cells and human macrophages. They also showed that recombinant PCSK9 induced TNFα mRNA in murine bone marrow-derived macrophages mainly, but not exclusively, in an LDLR-dependent fashion (28).

The primary source of local PCSK9 production in atherosclerotic plaques was reported to be smooth muscle cells (25), but other vascular cells such as endothelial cells (29) and macrophages (30) also express PCSK9 under inflammatory stimuli. Giunzioni et al. detected PCSK9 expression in murine macrophages and further reported that overexpression of human PCSK9 in macrophages induced the accumulation of Ly-6C^high^ monocytes in the atheroma and promoted atherosclerotic lesions in an LDLR-dependent mechanism, independently of blood lipid levels (31, 32). In contrast, deletion of the PCSK9 gene in the liver reduced atherosclerotic lesions without changing plasma cholesterol levels, primarily via LDLR-dependent mechanisms (33). These data suggest that PCSK9 exerts lipid-independent pro-inflammatory effects in an LDLR-dependent fashion.

## Lipid-independent and LDL receptor-independent pro-inflammatory effects of PCSK9

PCSK9 also targets other molecules than LDLR (e.g., very low-density lipoprotein receptor (VLDLR), apolipoprotein E receptor 2 (ApoER2), LDLR-related protein 1 (LRP1), CD36). Emerging evidence suggests that PCSK9 may have other targets associated with inflammation including the Toll-like receptor 4 (TLR4)/NF-κB signaling pathway and scavenger receptors.

The TLR4/NF-κB signaling pathway is one of the key signaling pathways mediating PCSK9-induced pro-inflammatory effects. PCSK9 silencing with lentivirus-mediated PCSK9 shRNA vector in *Apoe*^−/−^ mice ameliorated the development of atherosclerotic plaques associated with reduced number of macrophages and decreased expression of vascular inflammation regulators such as TNFα, IL-1β, MCP-1, TLR4 and NF-κB. Silencing PCSK9 did not affect the plasma lipid profiles in these mice. PCSK9 overexpression increased TLR4 expression and increased p-IkBα degradation, and NF-κB nuclear translocation in macrophages, but PCSK9 silencing had the opposite effects in RAW264.7 cells treated by oxidized LDL (oxLDL) (34). An important step in atherogenesis includes formation of ox-LDL that is taken up by a host of scavenger receptors such as SR-A, CD36, and LOX-1 on monocytes and macrophages (35). In an inflammatory milieu, PCSK9 stimulates the expression of scavenger receptors, principally LOX-1, and ox-LDL uptake in macrophages and thus contribute to the process of atherogenesis (30).

**Figure 3:**
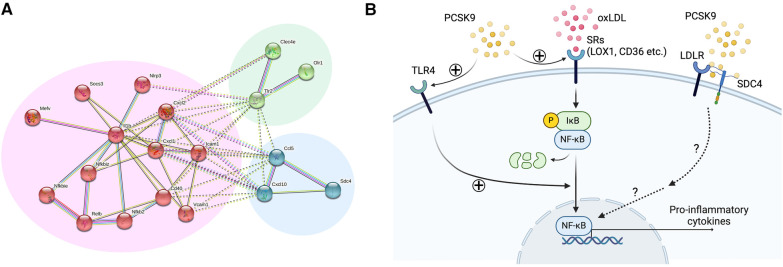
Lipid-independent and LDL receptor-independent pro-inflammatory effects of PCSK9 in macrophages. (**A**) Network analysis of the 24 overrepresented transcripts in *Ldlr*^−/−^ mouse macrophages using STRING database. Nodes are colored according to k-means clustering (number of clusters = 3). Only connected nodes are shown here. Panels were reproduced from Katsuki et al. with permission of the publisher. (**B**) Potential signaling pathways involved in PCSK9-induced macrophage activation. oxLDL binds to scavenger receptors (LOX1, CD36 etc.), leading to the phosphorylation and degradation of IκB and subsequent activation of NF-κB. PCSK9 promotes this process by enhancing scavenger receptor expression and TLR4. SDC4 directly binds to PCSK9, thereby inducing the expression of pro-inflammatory cytokines possibly via the NF-κB signaling pathway.

No previous *in vivo* studies, however, have provided direct evidence that demonstrates LDLR-independent pro-inflammatory effects of PCSK9 in macrophage activation and vascular lesions. We, thus, investigated LDLR-independent mechanisms by which PCSK9 induces pro-inflammatory activation of macrophages and accelerates vein graft lesion development using *Ldlr*^−/−^ mice. AAV vector encoding a gain-of-function mutant of PCSK9 (AAV-PCSK9) increased circulating PCSK9 without changing serum cholesterol and triglyceride levels (26). AAV-PCSK9 induced mRNA expression of the pro-inflammatory mediators IL-1β, TNFα, and MCP-1 in peritoneal macrophages, as underpinned by an *in vitro* analysis using *Ldlr*^−/−^ mouse macrophages stimulated with endotoxin-free recombinant PCSK9 (Figure 2). AAV-PCSK9 also promoted vein graft lesion development in experimental vein grafts of *Ldlr*^−/−^ mice. *In vivo* molecular imaging further demonstrated that AAV-PCSK9 increased macrophage accumulation and matrix metalloproteinase activity associated with decreased fibrillar collagen, a molecular determinant of atherosclerotic plaque stability. We then explored LDLR-independent pro-inflammatory signaling pathways in an unbiased manner, namely, a combination of unbiased global transcriptomics and network-based hyperedge entanglement prediction analysis. Potential targets of PCSK9 in macrophages include forementioned NF-κB signaling pathway and LOX-1, as well as a novel target, syndecan-4 (SDC4) (26). SDC4 is a heparan sulfate proteoglycan expressed on the surface of human macrophages (36). In addition, SDC4 mRNA is increased in bone marrow-derived macrophages stimulated with LPS, but not IL-4/IL-13 or IL-10 (37), suggesting a potential link between SDC4 and macrophage activation. Although Sdc4 was not a high-ranking differential expressed transcript when compared to NF-κB signaling molecules and Lox-1, its fold change after stimulation with recombinant PCSK9 in the transcriptomics was statistically significant. We performed *in vitro* experiments involving siRNA silencing in primary macrophages to substantiate our computational prediction platform and demonstrated that SDC4 indeed binds to PCSK9 *in vivo* and mediates PCSK9-induced pro-inflammatory responses. Understanding more detailed mechanisms by which SDC4 mediates the pro-inflammatory responses and their downstream signaling induced by PCSK9 requires future investigations (Figure 3). Collectively, we demonstrated LDLR-independent pro-inflammatory effects of PCSK9 in macrophage activation and vascular lesions, independently of serum lipid levels.

## Clinical application of PCSK9 inhibition in cardiovascular disease

Considering the role of PCSK9 in lipid metabolism, multiple therapeutic option including monoclonal antibodies (10, 11), small-molecule inhibitors (38), small interfering RNA (siRNA) molecules (15), antisense oligonucleotides (39), peptide vaccines (40, 41) and CRISPR/Cas9 editing (42) have been developed. While PCSK9 antibodies are administered biweekly, the frequency of PCSK9 siRNA administration is once every six months, substantially alleviating the burden on patients. As of today, the clinical impact caused by the distinction in the mechanisms of PCSK9 inhibition between PCSK9 antibodies and PCSK9 siRNA remains unknown. Elucidating this disparity requires further investigations. Nevertheless, patients with dyslipidemia eagerly await the development of orally administrable small molecular compounds since they are generally better tolerated than biotherapeutics, which require injection.

Over the past decade, we and other investigators have been exploring the potential for the development of a small molecule PCSK9 inhibitor. Miyosawa et al. from our group previously demonstrated that a new cholesteryl ester transfer protein (CETP) inhibitor, K-312, had dual inhibitory actions on CETP and PCSK9 (43). K-312 decreased the active forms of SREBP-1 and SREBP-2 by regulating the occupancy of SREBP-1 and SREBP-2 on the sterol regulatory element of the PCSK9 promoter. K-312 treatment attenuates atherosclerotic lesions in hyperlipidemic rabbits. Moreover, these K-312-treated rabbits possess decreased plasma levels of PCSK9, providing the *in vivo* evidence for the impact of this compound on PCSK9. Collectively, K-312 may serve as a potent add-on therapy to existing LDL-C lowering drugs such as statins. Merck & Co. has developed MK-0616, an oral PCSK9 inhibitor. In their Phase 2b trial, oral administration of MK-0616 at daily doses ranging from 6 mg to 30 mg resulted in significant reductions of plasma levels of LDL-C, surpassing those achieved with a placebo, in individuals with hypercholesterolemia with a broad spectrum of risks associated with atherosclerotic cardiovascular disease and background statin therapies. In addition, all doses of MK-0616 were well tolerated, with a minimal occurrence of serious adverse events and discontinuations due to adverse events, comparable to the placebo group. They have recently started a phase III trial of its oral macrocyclic peptide inhibitor of PCSK9 for atherosclerotic cardiovascular disease (38). As Rogers et al. from our group previously noted, the inhibition of DRP1 results in a reduction of hepatic PCSK9 secretion through the induction of hepatic proteasomal degradation of PCSK9 and the suppression of SREBP-1c (9). Consequently, an alternative therapeutic approach for PCSK9 inhibition involves a DRP-1 inhibitor, such as mdivi-1.

## Application of PCSK9 overexpression in basic research

Investigating therapeutic targets requires *in vivo* pre-clinical studies to demonstrate proof-of-concept. PCSK9 is not only a promising therapeutic target for hypercholesterolemia, but also applied in the development of experimental animal models for atherosclerosis. A single injection of an adeno-associated virus vector (AAV) encoding a gain-of-function mutant form of PCSK9, along with an atherogenic diet, induces atherosclerosis in mice and hamsters without genetic modification (44, 45).

Vascular calcification is one of the major clinical concerns. We typically use *Ldlr*^−/−^ or *Apoe*^−/−^ mice fed with a high-fat and high-cholesterol diet for 15–20 weeks to induce intimal calcification, a costly and time-consuming process. Recent studies have reported that PCSK9 transgenic mice (46) and D374Y PCSK9 transgenic pigs (47, 48) develop atherosclerotic lesions with calcification. We further reported that a single injection of D377Y PCSK9 AAV vector can induce experimental vascular calcification in mice (49). After a single injection of PCSK9 AAV to C57BL/6J mice fed a high-fat and high-cholesterol diet, serum levels of PCSK9 remained elevated for 20 weeks and vascular calcification progressed to the same extent as that in *Ldlr*^−/−^ mice. To validate the effectiveness of this experimental model of vascular calcification, our group demonstrated a reduction of aortic calcification in sortilin-deficient mice injected with PCSK9 AAV (50). Subsequently, we also demonstrated that DRP1 haplodeficiency did not alter aortic calcification using the same experimental approach (51). These data suggest that utilizing this experimental vascular calcification will enhance the efficiency of testing potential therapeutic agents for calcification *in vivo* without genetic manipulation to induce susceptibility to atherosclerosis.

## Summary and clinical perspective

Randomized clinical trials of PCSK9 inhibitors suggest that the lipid-lowering effect of PCSK9 monoclonal antibodies may be a major factor in the anti-inflammatory outcomes and reduction of cardiovascular events. However, the anti-inflammatory effects on vascular lesions of PCSK9 inhibitors, specifically in ameliorating macrophage activation beyond their lipid-lowering properties, remain largely unknown. In this review, we introduced a series of studies, including our own, that provide evidence supporting the hypothesis that PCSK9 may process pro-inflammatory properties independently of its lipid lowering effects. Part of the beneficial effects of PCSK9 inhibitors on cardiovascular morbidity and mortality in clinical trials might be attributed to a reduction in the low-grade inflammation. Potential future research directions include investigating whether PCSK9 inhibition can mitigate local inflammation in vascular lesions among patients, and elucidating the relative contributions of LDLR-dependent and independent mechanisms underlying the pro-inflammatory effects of PCSK9. In conclusion, the robust lipid-lowering effects of PCSK9 inhibitors with anti-inflammatory properties suggest their potential as a promising therapeutic option to treat cardiovascular diseases, including coronary artery disease, vein graft disease, ischemic stroke, peripheral artery disease, and other inflammatory disorders.
